# Study of the disintegration of loess modified with fly ash and Roadyes

**DOI:** 10.1038/s41598-023-33434-2

**Published:** 2023-05-04

**Authors:** Hongru Li, Min Yang, Xiaohan Guo

**Affiliations:** 1 Institute of Geotechnical Engineering, Xi’an University of Technology, No. 5, Jinhua South Road, Beilin District, Xi’an, Shaanxi China; 2 Loess Soil Mechanics and Engineering Key Laboratory of Shaanxi Province, Xi’an, China

**Keywords:** Ecology, Environmental sciences, Solid Earth sciences

## Abstract

The disintegration property of loess is the wetting and subsequent disintegration of loess in water, which is generally an important index for resistance to erosion and disintegration of wet loess slopes and foundations. In this study, a disintegration instrument is developed in this laboratory and used to study the disintegration properties of fly ash-modified loess in foundations and Roadyes-modified loess in subgrades. Disintegration tests are used to compare samples of loess modified with different amounts of fly ash and Roadyes, different water contents and different dry densities; the influence of fly ash and Roadyes content on the disintegration of modified loess is analyzed. The differences in disintegration properties between the pure loess and modified loess are compared to explore the evolution of disintegration properties of modified loess and the optimal incorporation levels of fly ash and Roadyes. The experimental results show that the incorporation of fly ash reduces the disintegration of loess, while the incorporation of Roadyes likewise decreases the disintegration of loess. The disintegration of the loess modified with the two curing agents is better than that of the pure loess and loess mixed with a single curing agent; the optimal incorporation levels are 15% fly ash and 0.5‰ Roadyes. Comparing the evolution of the disintegration curves of samples of loess with different modifications shows is a linear relationship between time and amount of disintegration for pure loess and Roadyes-modified loess. Thus, a linear disintegration model is established in which the parameter P is the disintegration rate. According to the exponential relationship between time and amount of disintegration of fly ash-modified loess and loess modified with both fly ash and Roadyes, an exponential disintegration model is established in which the water stability parameter Q affects the strong and weak disintegration of the modified loess. The relationship between the water stability of the loess (modified with added fly ash and Roadyes) in water and the initial water content and dry density is analyzed. The water stability of the loess first increases and then decreases with increasing initial water content and gradually increases with increasing dry density. When the sample density is the maximum dry density, the sample has the best water stability. These research results provide a basis for the application of loess modified with added fly ash and Roadyes.

## Introduction

The stability of loess is closely related to its water sensitivity. The water sensitivity of loess includes permeability, collapsibility, disintegration and erosion resistance^1,2^. Through theoretical analysis, experimental research and numerical simulation, many scholars have studied the macroscopic physical and mechanical properties of pure loess and modified loess from different aspects, such as the permeability^3–7^, collapsibility^8–10^ and erosion resistance properties^11–13^. Through scanning electron microscopy^14^, the influence mechanism of modified loess can be deeply understood from the change in microstructure. The disintegration of loess aggravates the development of loess gullies and karst caves and cuts or erodes a slope to cause slope instability, resulting in geological disasters such as collapses and landslides. The disintegration of loess also erodes building foundations, which causes collapses and ground subsidence. Therefore, it is important from a theoretical and practical standpoint to study the disintegration characteristics of loess in water and apply the results in soil conservation work and geotechnical engineering.

To improve the physical and mechanical properties of loess, common admixtures such as fly ash, lime, cement and fiber are typically employed^15^. Fly ash is an industrial waste with fine particles, stable chemical composition, large specific surface area, and certain activity. It is often used in combination with lime to improve strength. The incorporation of fly ash can improve the mechanical properties of loess^16–18^. Many scholars have conducted research based on the engineering properties of modified soil^19–21^ from the aspects of ratio^22^, mineral composition^23^ and microstructure^24^. However, there is a lack of research on the disintegration properties of fly ash-modified loess, and its wetting expansion and softening properties are unclear. There is an urgent need to study the disintegration properties of fly ash-modified soil.

Roadyes is a new soil curing agent (referred to as 3 s) that can significantly improve the dry density of loess by reducing permeability and improving shear strength. It has been widely used in the treatment of road subgrades, airport runways and other foundations. However, its influence on the disintegration properties of loess is unknown.

Research on the disintegration properties of loess has mainly involved the study of pure loess. Li et al.^25^ studied the two key factors of water content and compaction degree that affect the disintegration of loess in terms of microstructure. Li et al.^26^ analyzed the boundary effects of loess disintegration through laboratory and in situ disintegration tests of large specimens. Wang et al.^27,28^ studied the disintegration rate of Lishi loess through a laboratory water immersion test. Wang et al.^29^ studied the effect of alternating dry–wet conditions on the disintegration rate of soil by simulating dry–wet cycles with moisture. Gu et al.^30^ used a soil disintegration instrument made in their laboratory to study the factors that influence disintegration. These research results provide basic theories for the study of soil erosion, slope collapse and instability, but there is no research on the disintegration properties of loess modified with different added materials.

As the main solid waste discharged from coal-fired power plants, fly ash is widely used along with Roadyes to improve the physical and mechanical properties of loess. However, there are few studies on their influence on the disintegration properties of loess. As a substitute for lime and cement, Roadyes is combined with fly ash to change the disintegration of loess, which is a new approach. In this paper, a disintegration test instrument is designed in this laboratory and used to perform a quantitative comparison of disintegration tests involving pure loess and modified loess. The effects of fly ash alone or in combination with Roadyes on the disintegration properties of loess are explored, and the results provide a reference for the treatment of wetting expansion of foundations and slope erosion.

## Experimental program

### Materials

The loess is obtained from subgrade soil with a depth of 0.5–1.5 m at the Huazhou Avenue construction project in Weinan. The basic physical properties of the loess are verified in the laboratory and determined by the Proctor compaction test and liquid–plastic limit combined method. These parameters include a maximum dry density of 1.7 g/cm^3^, an optimal water content of 18.1%, a liquid limit of 29.8%, a plastic limit of 19.1%, a plasticity index of 10.7, and a natural moisture content of 8.7%. The fly ash is obtained from a thermal power plant in Zhengzhou, Henan. Roadyes, a new material for modified loess, is an ion soil stabilizer. It is made of a variety of ionic components by using polymer nanotechnology. Adding it to the soil allows the soil to consolidate under pressure. Roadyes is a new material jointly developed by Jiangsu Roadyes Construction Co., Ltd. and the Institute of Soil Science, Chinese Academy of Sciences. Roadyes received a national invention patent, and the specific composition is unknown.

### Test apparatus

**Figure 1:**
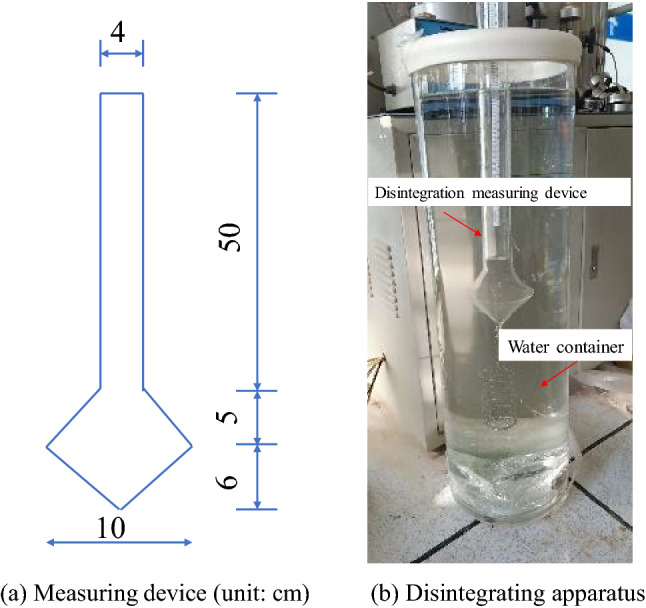
Disintegration instrument.

The test device is a set of disintegrating instruments designed and made independently on the basis of previous experience with soil disintegration. The instrument consists of a measuring device and a water container, as shown in Fig. 1. Figure 1a shows that the bottom of the measuring device is conical, the upper part is cylindrical, its interior can sustain a vacuum, and it is made of quartz glass. A removable barbed wire cylinder with an aperture of 1 cm and diameter of 4.5 cm is suspended at the bottom. The upper cylindrical part of the measuring device has a scale mark accurate to 0.1 cm, and the scale gradually increases from the bottom up. Figure 1b shows that the water container is a glass cylinder with a height of 1 m and a diameter of 30 cm.

The soil sample continuously disintegrates in the water and falls from the barbed wire cylinder, and thus the weight of the soil in the cylinder continuously decreases. The disintegration measuring device gradually floats in the water. The disintegration time–history curves of modified loess are drawn by reading the scale of the disintegration measurement device corresponding to the water surface in the water container at different times, which can reflect the characteristics of the disintegration process of the soil sample. The disintegration loss of the sample is calculated by Eq. (1).

1 $$W_{i} = \frac{{h_{1 - } h_{i} }}{{h_{1 - } h_{0} }}$$

where $W_{i}$ is the loss amount of disintegration of the sample at moment *i* (%), $h_{0}$ is the scale for the disintegration measuring device suspended in water by itself (m), $h_{1}$ is the scale at which the soil sample does not disintegrate after the sample is loaded into the disintegration measuring device and initially placed in water (m), and $h_{i}$ is the scale corresponding to the disintegration of the sample in water at moment *i* (m).

### Disintegration testing program

Modified loess samples with the optimal water content and maximum dry density are prepared with different curing agent contents. The influences of the dosages of fly ash and Roadyes on the disintegration properties of modified loess are studied. The dosage of fly ash or Roadyes is the mass ratio of fly ash or Roadyes to loess particles. The relationship of the two influencing factors on the disintegration characteristics of the modified soil is obtained by establishing the relationship between the disintegration amount of the sample and the time. The optimal dosage of the two curing agents is obtained when the water stability of the loess modified with added fly ash and Roadyes is the best. For the optimal dosage conditions, samples with different moisture contents and different dry densities are prepared to study the effects of the initial moisture content and dry density on the disintegration characteristics of modified loess. The testing program of the disintegration test is shown in Table 1.

**Table 1:** Testing program of disintegration test.

Variables	Values of variables
Incorporation level of the fly ash (%)	0, 10, 15, 20
Incorporation level of Roadyes (‰)	0, 0.5, 2.5, 5.0
Moisture content (%)	13, 15, 17, 18.94, 21, 23
Dry density (g/cm^3^)	1.4, 1.5, 1.6, 1.7

Three samples are prepared for each scheme combination to obtain the average disintegration time. The disintegration properties of modified loess are quantitatively analyzed by the correlation between disintegration loss and disintegration time.

## Disintegration properties of pure loess and modified loess

### Soil disintegration test

**Figure 2:**
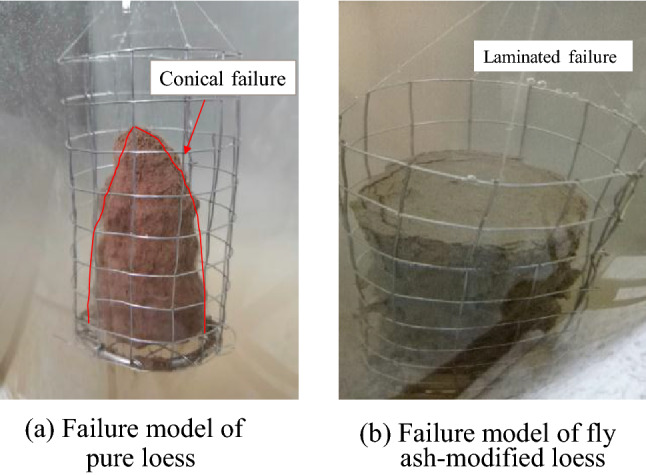
Destruction of pure loess and fly ash-modified loess.

The pure loess and fly ash-modified loess show an ‘inverted cone’ failure model of disintegration. When the fly ash-modified loess disintegrates in water, it shows a layered failure phenomenon from outside to inside, as shown in Fig. 2.

**Figure 3:**
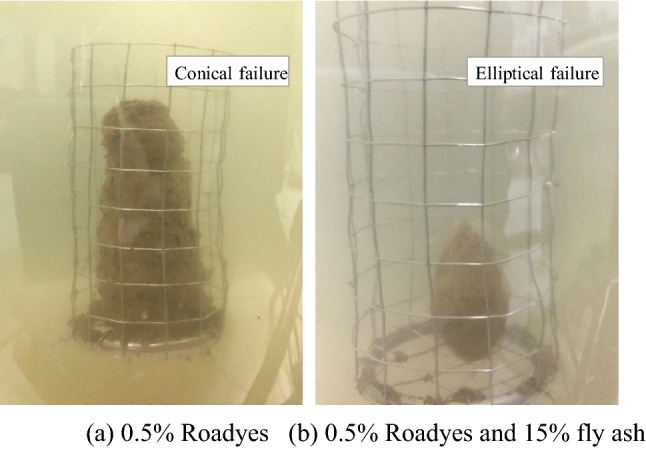
Disintegration and failure phenomena of soil samples with added fly ash and Roadyes.

The loess samples modified by addition of Roadyes show two kinds of failure phenomena: a ‘cone shape’ and an ‘ellipsoid shape’. The loess modified with added fly ash and Roadyes showed obvious ellipsoid failure characteristics during disintegration, especially the ellipsoid disintegration phenomenon, which was more obvious when the content of fly ash was large, as shown in Fig. 3.

Due to the weak cementation between particles of pure loess and fly ash-modified loess, the effect of gravity is obvious. When the erosive force of water destroys the cementation structure of the soil, the lower soil is subjected to the pressure (gravity) of the upper soil, which slows the disintegration rate, and the sample forms an ‘inverted cone’. After adding Roadyes to the fly ash-modified soil mixture, the chemical composition of the fly ash and the ions in the soil particles react with the Roadyes to form a very hydrophobic structure. The cemented structure is not easily destroyed. The sample is cylindrical. The edges formed on its side faces and base are eroded by water on both sides. This strong erosion force of water makes the two edges collapse first. As the edges gradually move to the interior of the sample, ellipsoid failure occurs.

The addition of fly ash has little effect on the failure characteristics of the sample. The addition of Roadyes changes the failure mode of the sample, which indicates that Roadyes has a significant effect on the water stability of the modified loess.

### Effect of fly ash content on disintegration characteristics of modified loess

**Figure 4:**
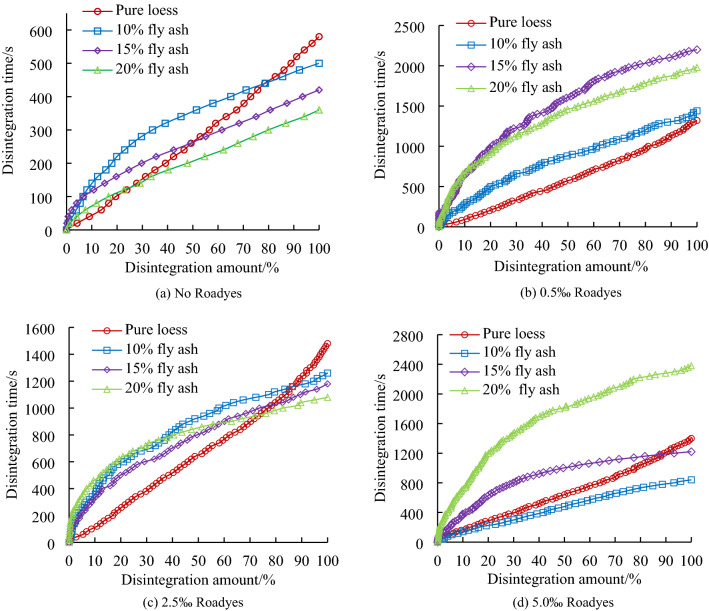
Disintegration and failure properties of soil samples with added fly ash and Roadyes.

The disintegration time–history curves of loess modified by adding different dosages of Roadyes and fly ash are arranged and analyzed, as shown in Fig. 4.

Figure 4 shows a linear relationship between disintegration time and disintegration amount of pure loess. When the disintegration amount of loess modified with added fly ash increases gradually, the disintegration time increases rapidly at first and then slows gradually. There is a nonlinear relationship between the disintegration time and the disintegration loss of the fly ash-modified loess. Figure 4a shows that the total disintegration time of the modified loess is less than that of pure loess when the fly ash-modified loess sample without Roadyes completely disintegrates. With increasing fly ash content, the disintegration time of modified loess decreases gradually; that is, the total disintegration times are ranked T_10%_ > T_15%_ > T_20%_ > T_0%_. When 0.5‰ Roadyes is mixed into the loess modified with the fly ash, the time to achieve the same amount of disintegration is longer than that of the loess modified with 0.5% Roadyes but without adding fly ash, and the disintegration time of the loess modified with 15% fly ash is the longest. The total disintegration times are ranked T_15%_ > T_20%_ > T_10%_ > T_0%_. When the Roadyes content is 2.5‰, the disintegration time of pure loess is the longest, and the total disintegration times are ranked T_0%_ > T_10%_ > T_15%_ > T_20%_. When the Roadyes content is 5‰, the disintegration time of the loess modified with 20% added fly ash is the longest, and the total disintegration times are ranked T_20%_ > T_0%_ > T_15%_ > T_10%_.

From the disintegration time–history curves of loess modified with fly ash, with increasing fly ash content, the time required for complete disintegration of the sample gradually shortens. The disintegration rate of the loess modified with added fly ash was initially higher than that of the pure loess. With increasing disintegration time, the disintegration rate of the modified loess with fly ash gradually decreased, while the disintegration rate of the pure loess gradually increased. The disintegration time–history curves with added Roadyes to the fly ash-modified soil mixture are complex, and they are related to the incorporation levels of fly ash and Roadyes. The fly ash particles are finer. The incorporation of fly ash increases the silt content of the soil and decreases the cohesive interactions between particles. The particle cementation of the modified soil mixed with only fly ash becomes weaker, resulting in lower strength and less resistance to water erosion, and thus the water stability worsens. The strength decreases, the ability to resist water erosion lessens, and the water stability worsens. The incorporation of Roadyes changes the disintegration characteristics of fly ash-modified loess. When the Roadyes content is 2.5‰, the disintegration time of the loess modified with 20% fly ash is the longest. The disintegration relationship of modified loess with different contents of road liquid and fly ash is not obvious.

### Effect of Roadyes content on the disintegration characteristics of modified loess

**Figure 5:**
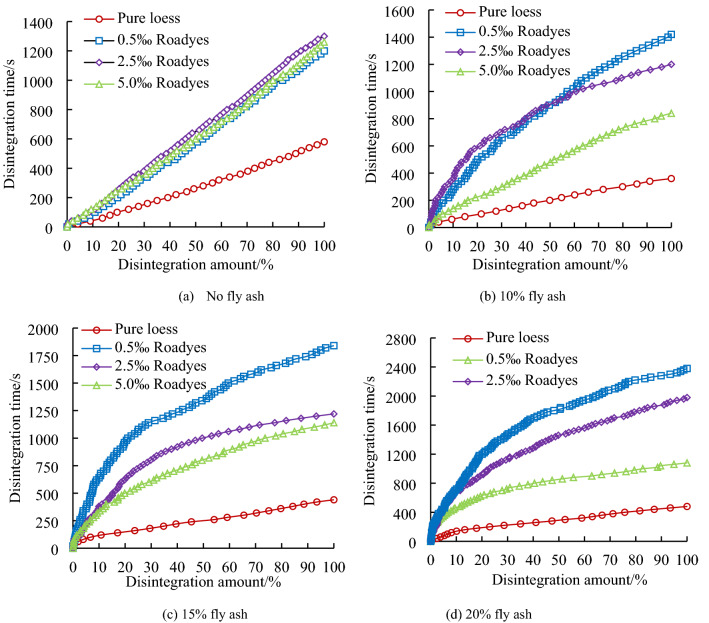
Disintegration properties of soil modified with different Roadyes contents.

The relationship curves of the disintegration amount of loess samples modified with different Roadyes and fly ash dosages vs. the disintegration time are shown in Fig. 5.

Comparing and analyzing the relationship curves in Figs. 4 and 5 shows that the disintegration curve of loess modified by adding different contents of only Roadyes has a linearly increasing relationship with the disintegration time, a longer disintegration time and better water stability. The complete disintegration times of samples with different amounts of Roadyes are basically the same and longer than that of pure loess. Figure 5 shows that when the content of fly ash is 10%, the disintegration time of loess modified with Roadyes is longer than that without Roadyes, and the total disintegration times of the samples are ranked T_0.5‰_ > T_2.5‰_ > T_5‰_ > T_0‰_. Before the disintegration amount reaches 50%, the disintegration rate of the Roadyes content of 2.5‰ is slower. When the disintegration amount exceeds 50%, the disintegration rate of the 0.5‰ Roadyes content is slower. The disintegration rate of loess modified with 5‰ Roadyes is faster than that with 2.5‰ and 0.5‰ Roadyes. When the fly ash content is 15%, the total disintegration times of the samples with different Roadyes contents are ranked T_0.5‰_ > T_2.5‰_ > T_5‰_ > T_0‰_. When the fly ash content is 20%, the total disintegration times of the samples with different Roadyes contents are ranked T_5‰_ > T_2.5‰_ > T_0.5‰_ > T_0‰_. The addition of fly ash weakens the disintegration of Roadyes-modified loess. When the content of fly ash is 15%, the disintegration of loess modified by fly ash and Roadyes is the weakest, and the disintegration time is the longest. The disintegration times of different Roadyes contents are different, which is explained by the optimal ratio of Roadyes and fly ash.

Analyzing the disintegration mechanism of Roadyes-modified loess shows the effect of Roadyes, which is a new material of soil stabilizer that uses polymer nanotechnology. A complicated reaction occurs between the polymer and the substances in the soil, and as a result the soil particles are “glued” together and provide water resistance. A three-dimensional thin shell solid matrix structure is generated between soil particles to form a semi-flexible solid matrix structure that changes the soil from hydrophilic to hydrophobic and improves the strength of the soil.

### Disintegration model of pure loess and Roadyes-modified loess

The influence of fly ash and Roadyes on the disintegration characteristics of modified soil is studied by analyzing the relationship between the disintegration amount of pure loess and modified loess with time. The disintegration amount and time has a linear relationship for pure loess and soil modified with the addition of Roadyes. The disintegration curves of fly ash-modified soil and mixed-modified soil show a nonlinear growth relationship.

A data analysis program is used to analyze the disintegration of pure loess and Roadyes-modified loess. The disintegration models of pure loess and Roadyes-modified loess are obtained as shown in Eq. (2).

2 $$T_{i} = P \cdot W_{i}$$

where $T_{i}$ is the total disintegration time of the sample at moment *i* (s), *P* is the test parameters obtained from the disintegration test, and $W_{i}$ is the disintegration amount of the sample at moment *i*, which ranges from 0 to 100.

The model parameter P reflect the disintegration rate of the sample, which is the time required for each 1% loss of the sample (s/%) to disintegrate. Therefore, the faster the disintegration rate index P is, the longer the time required for each loss of 1% mass of the sample. This indicates that the more difficult it is for the sample to disintegrate, the better the water stability of the sample.

The minimum value of the disintegration rate index P of the pure loess is 5.54 s/%, and the maximum value of the disintegration rate index P of the modified loess with 2.5‰ Roadyes is 13.52 s/%, which is approximately 2.5 times t of the disintegration rate P of pure loess by means of linear fitting. This indicates that the addition of Roadyes can enhance the water stability of soil, and the water stability of modified soil with only the addition of 2.5‰ Roadyes is the best.

The disintegration model parameters of pure loess and Roadyes-modified loess are shown in Table 2.

**Table 2:** Fitting parameters of the disintegration curves of pure loess and Roadyes-modified loess.

Incorporation rate of Roadyes (‰)	Disintegration rate/%	Correlation coefficient (R^2^)
0	5.540	0.9946
0.5	12.235	0.9900
2.5	13.521	0.9908
5.0	13.219	0.9937

The nonlinear curve fitting method is adopted for the disintegration curve of fly ash-modified loess and the combined modification loess of the Roadyes and the fly ash. The iterative calculation is selected as the orthogonal distance regression method. The fitting results show that the disintegration model of fly ash-modified loess and the combined modification of loess with Roadyes and fly ash conforms to the exponential growth relationship, and its calculation formula is Eq. (3).

3 $$T_{i} = - A \cdot \exp \left( { - W_{i} /B} \right) + C$$

where A, B and C are experimental parameters.

When the disintegration amount is 0, Eq. (3) reduces to Eq. (4):

4 $$Q = - A + C$$

The right side of Eq. (4) is a constant. Its geometric meaning is the intercept of the curve on the axis of the ordinate, and its physical meaning is the disintegration time when the disintegration amount of the sample is 0, which can be used to measure the time when the sample can maintain stability without disintegration in water. The parameter Q is used as an index to measure the water stability of fly ash-modified soil and the combined modification loess with Roadyes and the fly ash, and its unit is s.

The model parameters for loess disintegration with different fly ash and Roadyes contents are shown in Table 3.

**Table 3:** Parameters of the disintegration curve models of loess samples modified with fly ash and Roadyes.

Incorporation rate of the fly ash (%)	Incorporation rate of Roadyes (‰)	Estimating parameter			Water stability index (*Q*)/s	Correlation coefficient (R^2^)
		A	B	C		
10	0	544.76	56.40	584.37	39.61	0.9999
10	0.5	2213.58	111.20	2297.05	83.47	0.9998
10	2.5	1299.09	52.43	1425.97	126.88	0.9999
10	5.0	2417.24	236.29	2441.89	24.65	0.9999
15	0	599.07	104.88	642.15	43.08	0.9997
15	0.5	2352.97	50.88	2520.60	167.63	0.9999
15	2.5	1362.95	68.99	1474.72	111.77	0.9998
15	5.0	1211.76	33.16	1271.19	59.43	0.9999
20	0	789.36	182.11	809.97	20.61	0.9995
20	0.5	2032.38	53.79	2263.35	230.97	0.9999
20	2.5	947.57	41.40	1145.82	198.25	0.9992
20	5.0	2346.30	42.71	2573.99	227.69	0.9999

Table 3 shows that the nonlinear curve fitting method is adopted for the disintegration curve of fly ash-modified loess and the loess modified with the combination of Roadyes and the fly ash. All the correlation coefficients R^2^ of the fitted curves are greater than 0.99, which indicates a good fit of the curves.

**Figure 6:**
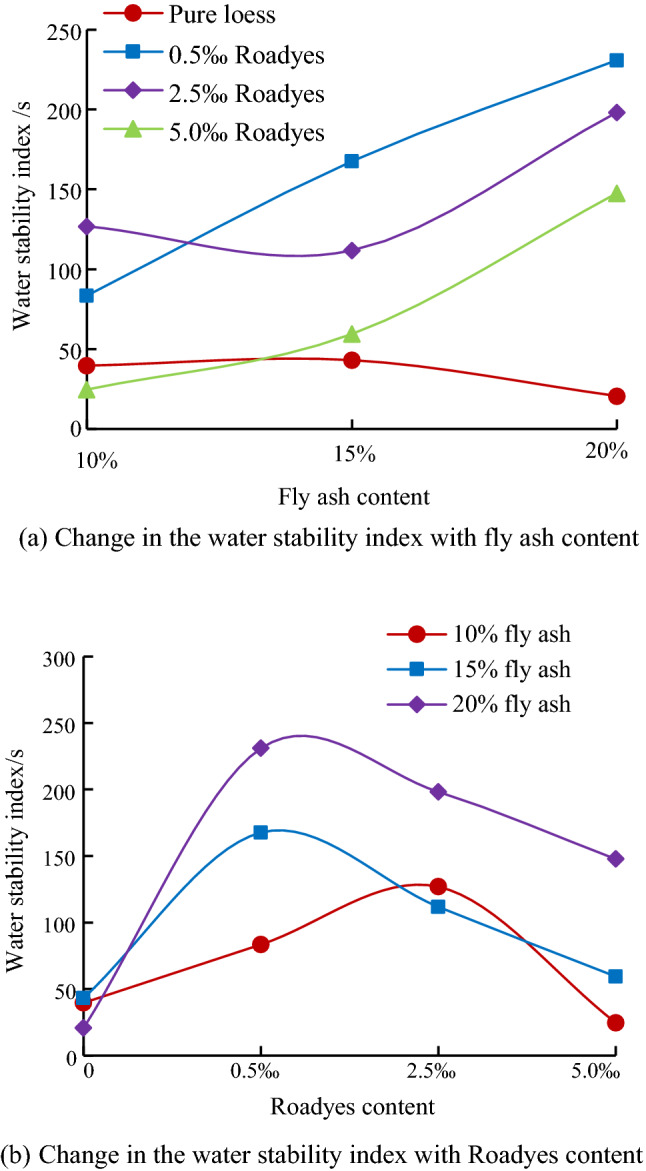
Change in the water stability index with curing agent dosage.

To directly reflect the variation rule of the water stability index Q, the results are analyzed to obtain the variation diagram of the water stability index Q with the content of fly ash and Roadyes, as shown in Fig. 6.

Figure 6a shows that when Roadyes is not added, the water stability index Q gradually decreases with increasing fly ash content, which indicates that the water stability of the soil gradually decreases. The water stability index Q of the combined modification loess with Roadyes and fly ash increases with increasing fly ash content. The addition of Roadyes significantly improves the water stability of fly ash-modified soil. Figure 6b shows that the water stability index Q increases first and then decreases with increasing amount of Roadyes for different fly ash contents. This shows that parameter Q can reasonably reflect the water stability of fly ash-modified loess and the loess modified with the combination of both additives.

Using the above model, the disintegration amount is set as 100%, and the time required for complete disintegration of the modified soil is obtained, as shown in Table 4.

**Table 4:** Parameters of the disintegration curve models of loess samples modified with fly ash and mixtures of additives.

Disintegration time (s)	Fly ash content			
	0	10%	15%	20%
Roadyes content				
0	554.00	491.86	411.27	354.15
0.5‰	1223.50	1396.42	1946.15	958.67
2.5‰	1352.10	1233.08	1154.85	1432.18
5.0‰	1321.90	858.74	1211.79	1904.29

## Influence of moisture content and dry density on disintegration characteristics of modified loess

By analyzing the disintegration characteristics of fly ash-modified loess and the combined modification loess, the optimal mass ratio of the two curing agents is obtained. During the test, it the disintegration characteristics of the modified loess are affected by the initial water content and dry density. Therefore, the combined modified loess with 15% fly ash content and 0.5‰ Roadyes content is taken as the research object to analyze the influence of the initial water content and dry density of soil on the disintegration characteristics.

### Influence of initial moisture content on disintegration characteristics of the sample

**Figure 7:**
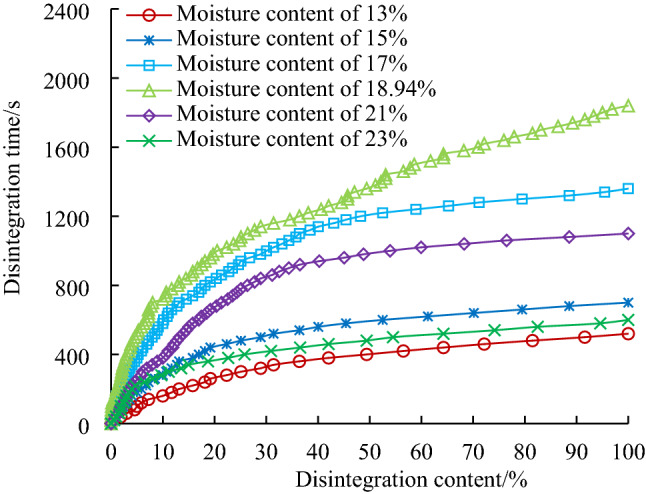
Disintegration curve of modified loess with different water contents.

Under the condition of a maximum dry density of 1.7 g/cm^3^, samples with different moisture contents are prepared, and the disintegration test results are shown in Fig. 7.

Figure 7 shows that the disintegration characteristics of the loess samples modified with both fly ash and Roadyes are closely related to the water content, and the disintegration amount is nonlinear with time. When the moisture content of the modified loess is closer to the optimal moisture content, its disintegration time is longer. When the moisture content of the sample is less than the optimal moisture content, the disintegration times of the samples are ranked T_13%_ < T_15%_ < T_17%_ < T_18.94%_, and the water stability of the sample gradually increases with increasing water content. When the water content of the sample is greater than the optimal water content, the disintegration times of the samples are ranked T_18.94%_ > T_21%_ > T_23%_, and the water stability of the sample gradually decreases with increasing water content.

### Influence of dry density on disintegration characteristics of samples

**Figure 8:**
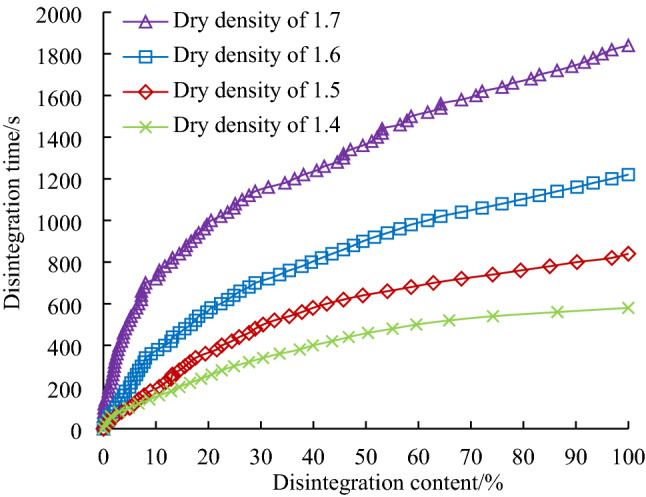
Disintegration curves of samples of modified loess with different dry densities.

Taking a fly ash content of 15% and a Roadyes content of 0.5‰ as the research objects, samples of modified loess with different dry densities are made under optimal moisture content conditions. The disintegration curves of loess with different dry densities are shown in Fig. 8.

Figure 8 shows that the disintegration amounts of the modified loess (15% fly ash, 0.5% Roadyes) with different dry densities have nonlinear relationships with time. The disintegration characteristics of the loess are closely related to the dry density of the samples. As the dry density gradually increases to the maximum dry density, the water stability of the modified loess gradually increases. When the loess is completely disintegrated, the disintegration times for different dry densities are ranked ${\text{T}}_{{1.7\,{\text{g/cm}}^{3} }} > {\text{T}}_{{1.6\,{\text{g/cm}}^{3} }} > {\text{T}}_{{1.5\,{\text{g/cm}}^{3} }} > {\text{T}}_{{1.4\,{\text{g/cm}}^{3} }}$. The dry density has a significant effect on the disintegration characteristics of the loess modified with 15% fly ash and 0.5% Roadyes.

## Discussion

The disintegration properties of loess are related to cementation force between its particles in water. Cementation is the material basis of disintegration. The weakening of the cementation force between soil particles is slight in water, and thus the loess collapses slightly. When the cementation force decreases rapidly in water, the connection force between soil particles is lost, and the loess disintegrates. The strength of the cementation force between loess particles depends largely on the material composition of the cement. Different cements show different properties in water, which affects the cementation force.

In this paper, the disintegration properties of loess modified with added fly ash and Roadyes are studied. The incorporation of fly ash and Roadyes into loess mainly changes the characteristics of cementing material between soil particles. The glass microsphere particles of fly ash in the clay play a ‘lubrication’ role and are dispersed in clay, which reduces the friction between soil particles and makes loess more prone to compression and densification. The compactness of the soil makes it difficult for water to penetrate the soil, slowing the disintegration process. The effect of Roadyes on loess mainly has three aspects. First, the soil particles are subjected to charge exchange to neutralize the negative charge on the surface of the soil particles and thus the soil particles lose their electrostatic attraction to water, thereby replacing the bound water around the soil particles with free water and permanently changing the water absorption of the soil. Second, under the catalysis of Roadyes, the chemical composition of the soil generates hydrated silicic acid gel, which acts as a lubricant, making the soil easier to compact and achieve higher compactness. Third, after the soil is compressed, the subcapillary pores less than 0.002 mm are filled by the copolymer colloidal clay particles produced by the reaction between Roadyes and the material in the soil, which blocks the capillary water flow channels that control the permeability of the loess. Therefore, when the Roadyes-modified loess is immersed in water, the Roadyes prevents the water from penetrating the loess and slows the disintegration of loess.

In other studies, an appropriate amount of polypropylene fiber was incorporated into loess^15^. At the macroscopic level, the physical restraint provided by polypropylene fiber was used to improve the disintegration resistance characteristics of loess, which were completely different from those of loess modified by fly ash and Roadyes. All studies focused on pure loess^25,26,28,30^, and the influences of soil sample size, boundary conditions, pore size, water temperature, natural water content and compaction degree on the disintegration characteristics of loess were studied. These studies were different from this study where the disintegration characteristics of loess modified by two curing agents, fly ash and Roadyes, were explored. The incorporation of a curing agent changes the structure of loess on the microscopic level to improve its disintegration resistance characteristics. Using chemical substances to change the physical and mechanical properties of loess has great application prospects.

## Conclusions

The disintegration characteristics of loess improved with added fly ash and Roadyes were studied by using a disintegration instrument designed in this laboratory. The effects of curing agent dosage, moisture content and dry density on the disintegration characteristics of modified loess were analyzed. The following conclusions were drawn:

1. The addition of fly ash changed the disintegration resistance of pure loess and reduced the disintegration time. The addition of two curing agents, Roadyes and fly ash, improved the water stability of modified loess, and the water stability was better than that of pure loess and loess modified with the addition of a single curing agent.
2. The optimal incorporation ratios of fly ash and Roadyes were 15% and 0.5‰, respectively.
3. A linear disintegration model of loess modified with the addition of Roadyes was established, in which parameter P evaluated the disintegration rate of loess. According to the disintegration characteristics of loess modified with added fly ash and loess modified with both fly ash and Roadyes, an exponential disintegration model was established in which the water stability index Q was used to evaluate the water stability of modified loess.
4. According to the total disintegration times of samples of modified loess, the water stability of loess modified with the optimal dosages of fly ash and Roadyes was approximately 3.5 times greater than that of the pure loess.
5. When the moisture content of loess modified by fly ash and Roadyes was less than the optimal water content, the water stability gradually increased with increasing water content. When the water content of the loess modified by fly ash and Roadyes was greater than the optimal water content, the water stability decreased gradually with increasing water content, and the decrease was larger. As the dry density of the modified loess gradually increased to the maximum dry density, the water stability of the sample gradually increased.

## Data Availability

The data used to support the findings of this study are included within the article.

## References

[1] Liu Z (1997). Mechanics and Engineering of Loess.

[2] Xie D (2015). Soil Mechanics for Unsaturated Soils.

[3] Xu PP, Zhang QY, Qian H, Li MN, Yang FX (2021). An investigation into the relationship between saturated permeability and microstructure of remolded loess: A case study from Chinese Loess Plateau. J. Hydrol..

[4] Xu PP, Qian H, Zhang QY, Zheng L (2021). Exploring the saturated permeability of remolded loess under inorganic salt solution seepage. Eng. Geol..

[5] Zhang YT, Qian H, Hou K, Qu WG (2021). Investigating and predicting the temperature effects of permeability for loess. Eng. Geol..

[6] Xu PP, Qian H, Zhang QY, Shang JT, Guo YK, Li MN (2022). Response mechanism of permeability change of remolded loess to seepage parameters. J. Hydrol..

[7] Xiao DH, Feng WJ, Zhang Z, Ming J (2015). Research on the relationship between permeability and construction feature of loess under the freeze-thaw cycles. China Hydrogeol. Eng. Geol..

[8] Yang H, Xie WL, Liu QQ, Zhu RS, Liu YY (2022). Three-stage collapsibility evolution of Malan loess in the Loess Plateau. CATENA.

[9] Yao YG, Zhang YC, Gao XL, Huang HW, Liu DF, Hui XQ (2021). Study on permeability and collapsibility characteristics of sandy loess in northern Loess Plateau, China. J. Hydrol..

[10] Zheng ZY, Li XA, Wang L, Li LC, Shi JF, Bi ML (2021). A new approach to evaluation of loess collapsibility based on quantitative analyses of colloid-clay coating with statistical methods. Eng. Geol..

[11] Sun XH, Miao LC, Chen RF, Wang HX, Xia JX (2021). Surface rainfall erosion resistance and freeze-thaw durability of bio-cemented and polymer-modified loess slopes. J. Environ. Manag..

[12] Cheng YJ, Tang CS, Pan XH, Liu B, Xie YH, Cheng Q, Shi B (2021). Application of microbial induced carbonate precipitation for loess surface erosion control. Eng. Geol..

[13] Zhang HG, Liu GQ, Zhao CS, Zhang LH, Zhang Q, Fu H, Cao S (2021). Loess erosion change modeling during heavy rainfall. Int. J. Sediment Res..

[14] Liu ZZ, Wang Q, Zhong XM, Liu FQ, Liang SY (2020). Water holding capacity and water stability of lignin-modified. Chin. J. Rock Mech. Eng..

[15] Lu H, Yan C, Jia ZL, Lan HX, Shi YL, Yang XH, Zhang ZQ (2021). Shear strength and disintegration properties of polypropylene fiber-reinforced loess. J. Traffic Transp. Eng..

[16] Li M, Wang C, Du HP, Zhang JX (2019). Mechanical properties of oil contaminated saline soil solidified with lime and fly ash. China J. Rock Mech. Eng..

[17] Yang YH, Liang B, Ding L (2001). Experimental study on the strength behaviors of flyash-lime or flyash-cemen. China J. Geotech. Eng..

[18] Wang XC, Li ZX, Xue H (2007). Study of sub-base performance of low liquid limit silt stabilized by lime and fly ash. China Eng. J. Wuhan Univ..

[19] Luo YS, Li J, Xu L (2009). Study of engineering property of mixed-soil fly ash. China Rock Soil Mech..

[20] Guo H, Luo YS, Yang YJ (2009). Permeability characteristic of fly ash added by loess. China J. Northwest A&F Univ. (Nat. Sci. Ed.).

[21] Yang AW, Xiao M, Zhou YM (2019). Experimental Study on lime-fly ash to cure tianjin marine soft soil. China J. Undergr. Space Eng..

[22] Xia Q, Yang YH, Geng X (2008). Experimental study on fly ash-lime or fly ash-cement loess filling. China J. Lanzhou Jiaotong Univ..

[23] Meng SJ, Zhang HR, Wang M, Sun YQ, Kuang Y (2019). Experimental study of mechanical properties for hydraulic fill sand and fly ash composite soil. China J. Nat. Disasters.

[24] Ji GD, Yang CH, Liu W, Zuo JJ, Lei GW (2015). An experimental study on the engineering properties of backfilled alkali wastes reinforced by fly ash. China Rock Soil Mech..

[25] Li JC, Tian WP (2005). Experiment of compacted loess disintegration. China J. Chong Qing Jiao Tong Univ..

[26] Li XA, Huang RQ, Peng JB (2009). Experimental research on disintegration of loess. China J. Rock Mech. Eng..

[27] Wang NQ, Wei JR (2015). Development and application on the instruments of constant volume loess disintegration. China J. Geol. Hazard Control.

[28] Wang NQ, Wei JR (2015). Experimental study on disintegration rate of Lishi loess in Guanzhong region of Shaanxi province. China J. Eng. Geol..

[29] Wang J, Ma F, Zhang PH, Meng QQ, Zhang QF (2015). Effect of wet-dry alternation on loess disintegration rate. China Acta Pedol. Sin..

[30] Gu TF, Yuan L, Hu W, Zhu LF, Wang X (2017). Experimental research on disintegration of the Heifangtai loess. China Hydrogeol. Eng. Geol..

